# Effect of Surface Morphology and Internal Structure on the Tribological Behaviors of Snake Scales from *Dinodon rufozonatum*

**DOI:** 10.3390/biomimetics9100617

**Published:** 2024-10-11

**Authors:** Ge Shi, Jinhao Wang, Yuehua Dong, Song Hu, Long Zheng, Luquan Ren

**Affiliations:** 1 Key Laboratory of Bionic Engineering (Ministry of Education), Jilin University, Changchun 130025, China; 18643114661@139.com (G.S.); wangjinhao0533@163.com (J.W.); 18332580119@163.com (S.H.); lqren@jlu.edu.cn (L.R.); 2 Weihai Institute for Bionics, Jilin University, Weihai 264402, China; huad0511@163.com

**Keywords:** bionic materials, microstructure, morphology, tribological behaviors, sliding locomotion

## Abstract

Snakes can move freely on land, in lakes, and in other environments. During movement, the scales are in long-term contact with the external environment, providing protection to the body. In this study, we evaluated the mechanical properties and scratching performance of the ventral and dorsal scales from *Dinodon rufozonatum*, a generalist species that moves on both land and in streams under wet and dry conditions. The results showed that the elastic modulus and hardness of the dry scales were greater than those of the wet scales. The average scale friction coefficient under wet conditions (0.1588) was 9.3% greater than that under dry conditions (0.1453). The scales exhibit brittle damage in dry environments, while in wet environments, ductile damage is observed. This adaptation mechanism allows the scales to protect the body by dissipating energy and reducing stress concentration, ensuring efficient locomotion and durability in both terrestrial and aquatic environments. Understanding how this biomaterial adapts to environmental changes can inspire the development of bionic materials.

## 1. Introduction

Biological materials have evolved various mechanical properties over millions of years of natural evolution, inspiring bionic engineering [1,2,3]. A challenging area in the field of bionic science is the adaptability of natural biomaterials to environments and their unique properties [4,5,6]. These materials exhibit a wide range of properties, including structural hierarchies, mechanical resilience, and adaptability to environmental conditions, which are essential in bioinspired material design [7,8]. For instance, studies on reptiles, particularly snakes, have revealed how surface texture and material anisotropy contribute to their locomotion and environmental adaptation [9]. A challenging area in the field of bionic science is the adaptability of natural biomaterials to environments and their unique properties. In particular, the ability of snakes to move efficiently across different terrains offers potential bioinspiration for advanced materials with tunable friction and wear properties [10,11]. Squamates occupy habitats ranging from tropical oceans to temperate mountaintops, making them the most diverse and species-rich group of living reptiles [12]. Squamate reptiles (approximately 10,000 species of snakes and lizards) are a diverse group of terrestrial animals. The diversity of this biological group presents a significant possibility for bioinspired solutions to a wide range of contemporary technological issues, particularly in the fields of surface engineering and tribology [13]. Scale, a natural material covering the entire body of squamate reptiles, has received considerable research attention [14,15,16,17,18,19]. Bionomic studies at various scales have enabled a variety of engineering applications, including surface texture [7,13,20,21,22,23,24,25], internal structure [26,27,28,29,30,31], chemical composition [14,30,32,33,34,35], mechanical properties [3,29,30,32,36,37,38,39], and friction properties [7,22,23,24,26,40,41,42,43]. In particular, snake scales exhibit outstanding frictional properties, permitting effective limbless movement in complex living environments [23,24,40,41].

Four major forms of terrestrial progression have been identified in snakes: (I) serpentine movement, which is observed in virtually all genera; (II) concertina movement; (III) crotaline or “side-winding” movement; and (IV) rectilinear movement, as observed in boas and other large species [44]. The vast regions of the snakeskin are crucial for transferring locomotor forces. Snakes frequently have overlapping scales that are thicker and stiffer than the skin at the hinge areas between scales. Therefore, the skin can frequently expand considerably without exposing the hinge area to the surfaces on which the snake crawls. Limbless locomotion is mainly driven by the highly frictional interface between the ventral surface of the animal and the base when the body bends. Previous studies revealed two distinct origins of a developed, nearly isotropic, pitted ventral micro-texture in three viper species that correlate with sidewinding locomotion specialization, indicating adaptation [45]. Ventral scales are often macroscopically more uniformly smooth, although their microscopic structure can contribute to a directionally dependent frictional resistance [46]. Scholars’ estimates of body speed indicate that snake propulsion on flat ground, and probably in general, is dependent on the frictional anisotropy of their scales [47].

Snake scales serve a variety of functions, including body protection [14,48,49], temperature adjustment [50,51], water loss reduction [35,39], radiation protection [51], and color display [48,51,52,53]. There is a special six-layer structure in snake scales, including the Oberhäutchen, β-layer, meso-layer, α-layer, lacunar tissue, and clear layer [24,27,54,55]. Like many biological organisms, snake scales have a gradient of material properties; the outermost layer is hard and inflexible, while the inner layer is soft and flexible [9,56]. As Stanislav N. Gorb points out in his argument, the outer scale layers have a higher effective elastic modulus than the inner scale layers [54]. Until recently, most of the research has been performed on the ventral scales, but there have been few studies on the mechanical properties of the dorsal scales under both dry and wet conditions.

This study aimed to determine the mechanical and scratch properties of snake scales in a variety of environments and to assess their adaptability to these environments. The mechanical properties of the ventral and dorsal scales of *Dinodon rufozonatum*, including creep, elastic modulus, and hardness, were tested under dry and wet conditions. Scratching experiments were conducted to determine the friction coefficient of the scales, and the microstructure of the scales after scratching was observed to determine the primary failure mechanisms of the scales in different situations and the environmental adaptability they displayed.

## 2. Materials and Methods

### 2.1. Animals and Preparations

**Figure 1:**
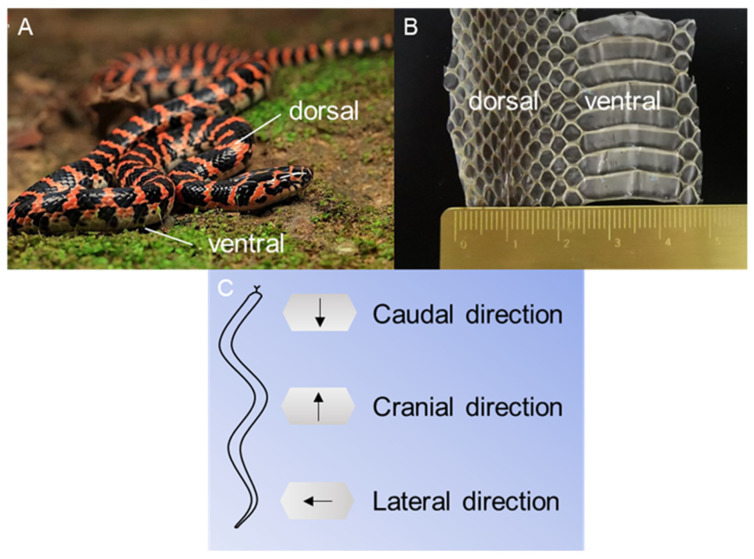
The ventral and dorsal scales of *Dinodon rufozonatum* (**A**) and the exuvium (**B**). The arrows in the scratch directions of the scales (**C**) indicate the displacement direction of the scratch head relative to the scale during the scratch test.

*Dinodon rufozonatum* (Figure 1) is a snake species that is primarily found in China. It inhabits a diverse range of habitats, including hills, mountains, plains, fields, villages, and bodies of water. Because the snake epidermis is composed of dead cells and cellular derivatives, the differences between the exuvium and the skin of living snakes are minimal [57,58]. Furthermore, the shed epidermis was the most reliable specimen since it posed no danger to the snakes. The skin samples used in the experiment were obtained naturally from snakes. The exuvium of three *Dinodon rufozonatum* snakes, weighing 0.21 ± 0.07 kg and measuring 1.04 ± 0.21 m in length, was used in this study. There were 187 ventral scales on the snake skin, and the force on each was approximately 11.2 mN. We divided the shed scales into three parts according to the average length of the snake from the snout to the anus and selected 5–30%, 30–55%, and 55–80% of the total length of the snake as the scales in the head, middle, and tail regions, respectively.

The snake scales were carefully cleaned for 15 min with an ultrasonic cleaner (K× −1840 T, KESH Co., Ltd., Beijing, China), after which the excess water was blotted with absorbent paper. Dry scales were adhered flat to the glass substrates using double-faced adhesive tape (Changdasheng Electronics Co., Ltd., Shenzhen, China) to eliminate bubbles, wrinkles, and other defects. For wet conditions, the scales were hydrated in distilled water for 30 min before being adhered flat to the substrates with double-faced adhesive tape using a light plastic tweezer to avoid damage to the scales.

To assess the mechanical properties of the snake scales, a minimum of twelve specimens were prepared for each condition, for a total of 72 specimens (two types of scales (ventral and dorsal) × three locations (head, middle, and tail) × two moisture conditions (dry and wet) × 6 specimens = 72). All the tests were carried out in a controlled laboratory environment (25 °C).

### 2.2. Atomic Force Microscopy (AFM)

The surface morphology of the scales was obtained by AFM (Tosca 200, Anton Paar Company, Graz, Austria) in tapping mode. The scanning range was 10 μm × 10 μm, and the scans were carried out with a scan rate of 1 Hz and a resolution of 400 × 400 pixels using a ScanAsyst-air cantilever. The height of the microstructure was estimated from AFM images processed by Tosca Analysis software 7.4 (Tosca 200, Anton Paar Company, Graz, Austria).

### 2.3. Nanoindentation Test Procedure

The mechanical properties of the scales were determined using a nanoindentation tester (Step 500 NHT3, Anton Paar Company, Graz, Austria) equipped with a Berkovich trigonal pyramid indenter at an angle of 142.3°, and the Poisson’s ratio of the samples was set to 0.3. The indentation experiments were performed using a conventional trapezoidal loading scheme, including a 30 s loading period, a 10-s hold at a maximum loading force of 2 mN, and a 30-s unloading period. Moreover, the sinus mode was adopted on the scales with a 0–20 mN load range, a sinus amplitude of 2 mN, and a loading rate of 0.1 s^−1^. The creep, elastic modulus, and hardness of each specimen were determined from the force and displacement curves using the Oliver-Pharr method. The mechanical properties were statistically analyzed by using ANOVA and Tukey’s HSD tests, with *p* < 0.05 indicating statistical significance. All analyses were performed with SPSS version 25.0 software (SPSS Inc., Chicago, IL, USA). Each sample was measured at least five times to obtain a mean value and standard deviation. The scales were approximately 30 μm thick. According to the indentation principle, all indentation experiments were conducted with a maximum indentation depth of less than 10% of the sample thickness to eliminate the effect of the sample substrate on the experimental results [9,59].

### 2.4. Scratch Test Procedure

The scratch measurements were performed on a Micro Scratch Tester (Step 500 MST3, Anton Paar Company, Graz, Austria) with a spherical Rockwell indenter with a radius of 100 μm. Each sample was scratched nine times with constant forces of 50, 100, and 200 mN at a linear speed of 2 mm/min and a stroke of 1 mm. All scratches were made from head to tail under both dry and wet conditions. In addition, a constant normal force of 200 mN was used to test the scales for scratching in three directions: caudal, cranial, and lateral. Nine tests were performed under each condition. Once the scratch test is completed, the average coefficient of friction of the scales in each condition is obtained so that the friction performance of the scales in different places and under wet and dry conditions can be compared. After obtaining the friction coefficients, statistical analysis was performed using SPSS version 25.0 software (SPSS Inc., Chicago, IL, USA) and Origin version 2018 software (OriginLab, Northampton, MA, USA).

We applied a progressive load to the ventral and dorsal scales of the snake. The sliding speed was set to 2 mm/min, the scratch length was set to 1 mm, and the loading range was set to 50–3000 mN. Under dry and wet conditions, all progressive force scratches were carried out in three directions on each sample, namely, caudal, cranial, and lateral (Figure 1), and the samples were examined after scratching using a scanning electron microscope.

### 2.5. Scanning Electron Microscopy (SEM)

The samples were sputter-coated with gold-palladium (10 nm thickness) in a vacuum environment using an SBC-12 Ion Sputter Coater (KYKY Technology Co., Ltd., Beijing, China) to improve imaging quality before being retested. After that, the micromorphology and quality of the abrasion traces of the scales were examined using a LYRA3 scanning electron microscope (TESCAN ORSAY HOLDING, a.s., Brno, Czech Republic) at an accelerating voltage of 5 kV. To obtain cross-sectional images of the scales, the samples were frozen in liquid nitrogen and fractured, and the above steps were repeated.

### 2.6. Raman Spectroscopy (RS)

A Raman microscope (DXR3, Thermo Fisher Scientific, Waltham, Massachusetts, USA) was used to obtain Raman images of the scales. The system was equipped with a diode laser with 532 nm incident light, a 10× Olympus objective lens (N.A. = 0.4), and a motorized stage to allow micro-positioning of small tissue samples. A single 900 lines/mm grating was used to disperse the collected light, and a charged coupled device detector measured the signal at each wavelength. Raman intensity and Raman shifts were acquired with a continuous laser beam with an accumulation time of 0.5 s per point and a pre-bleaching time of 0.5 s. All background signals were corrected by a multipoint baseline, and peak positions were subsequently determined. OMNIC for dispersive Raman spectroscopy and OriginPro 2021 software (OriginLab Corporation, Northampton, MA, USA) were used for image processing and analyses.

### 2.7. Tribological Testing of Biomimetic Surfaces

A textured surface mimicking snake scales was created on spheroidal graphite cast iron using a COHERENT AVIA355-14 laser (wavelength 354.7 nm). The cast iron samples (15 mm × 10 mm × 5 mm) were paired with a 304 stainless steel pin (3 mm diameter) for friction testing. After laser processing, the textured surfaces were polished using 2000-grit sandpaper to remove slag, followed by diamond paste (W1.0) polishing to achieve a surface roughness of 0.2 μm. The same polishing was applied to the steel pin. Friction and wear tests were performed on a tribometer (Rtec Instruments, MFT-5000) under dry conditions, with a normal load of 4 N, sliding speed of 10 Hz, and a stroke length of 10 mm. The tests lasted 50 min:15 min for running-in wear and 35 min for steady-state friction. Each sample underwent three parallel tests to minimize random error. Wear volume was determined by measuring sample mass before and after testing, and the friction coefficient was recorded for analysis.

## 3. Results

### 3.1. Morphology and Structure of Scales

**Figure 2:**
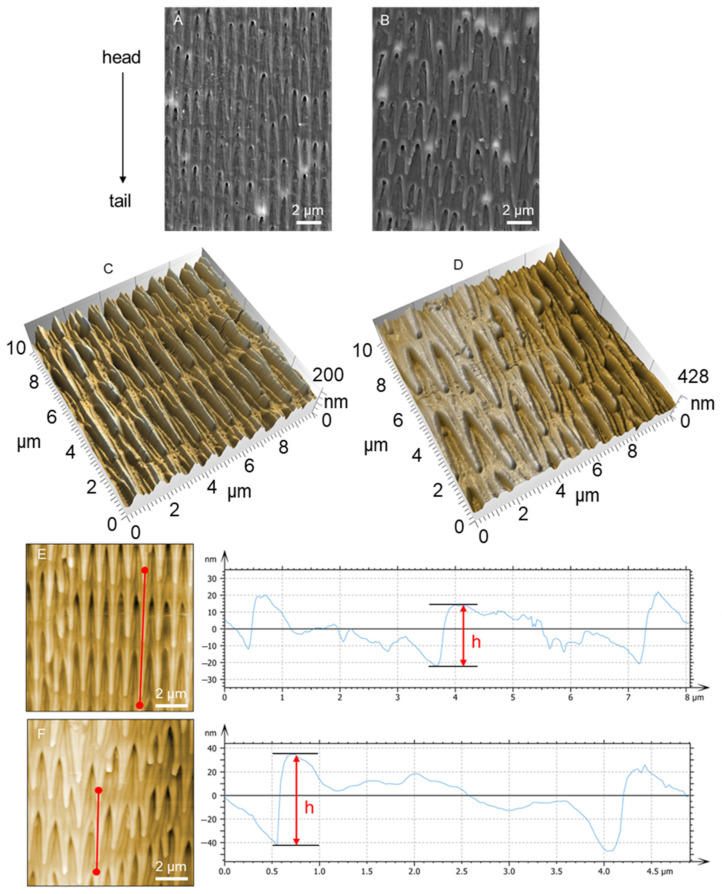
SEM images of the surface morphology of the ventral (**A**) and dorsal (**B**) scales at 15k× magnification. Three-dimensional topography of the scale surface for the ventral (**C**) and dorsal (**D**) scales from AFM profiling. The heights of the microstructures of the ventral (**E**) and dorsal (**F**) scales are indicated by red lines.

As shown in Figure 2, the surface morphology of the ventral and dorsal scales was determined by SEM (Figure 2A,B) and AFM (Figure 2C,D). Many three-dimensional microstructures were oriented on the surface of the ventral and dorsal scales. In the direction parallel to the scale surface, the microstructures looked like nails and were arranged in a comb-like pattern. The orientation of the microstructure tips was toward the snake tail. However, in the direction perpendicular to the scale surface, the microstructures were similar to those of the sawtooth and were arrayed in parallel lines. The orientation of the microstructure tips was also biased toward the snake tail. The 3D microstructure and shape of the scales play a crucial role in their tribological behavior. The sawtooth-like microstructure on the ventral scales, oriented toward the tail, contributes to directional friction modulation. The shape ensures minimal friction in caudal motion while maximizing resistance in lateral and cranial movements. SEM images further support this, showing how the microstructure deforms differently under various scratch directions, thus explaining the scales’ anisotropic frictional properties. The heights of the microstructures are indicated in Figure 2E,F. The length, width, and height of the microstructures were 2–5 μm, 0.5–1 μm, and 30–80 nm, respectively. Compared to those on the dorsal scales, the microstructures on the ventral scales were regular and uniform. Moreover, the size and space of the microstructures on the ventral scale were smaller than those on the dorsal scale, for which the ventral scale microstructures were arranged more densely (Figure 2A).

**Figure 3:**
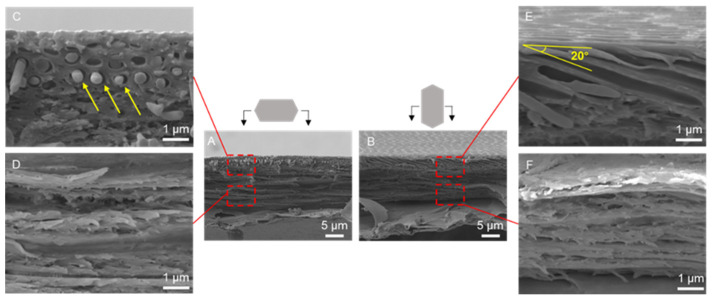
SEM images of the cross-sectional structure at the ventral scale at 5k× (**A**,**B**) and 30k× (**C**–**F**) magnification. The yellow arrows point to the fibers.

The cross-sectional morphology of the ventral scale is displayed in Figure 3. The ventral scale had a thickness of approximately 25 μm (Figure 3A,B) and was composed of an upper fibrous (Figure 3C,E) layer and a lower lamellar layer (Figure 3D,F). The fibrous layer was approximately 4 μm thick and comprised many fibers. The fibers were approximately 0.5 μm in diameter (Figure 3C) and tilted to the scale surface at an angle of approximately 20° (Figure 3E). Below the fibrous layer, there was no visible fibrous tissue in the lamellar layer. The lamellar tissues were tightly stacked, with spaces between them (Figure 3D,F).

**Figure 4:**
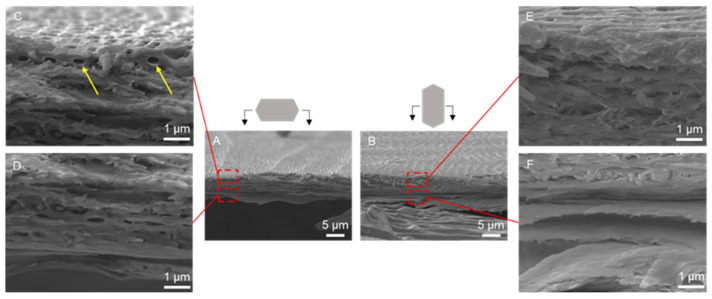
SEM images of the cross-sectional structure of the dorsal scale at 5k× (**A**,**B**) and 30k× (**C**–**F**) magnification. The yellow arrows point to the fibers.

The cross-sectional morphology of the dorsal scale is displayed in Figure 4. The dorsal scale was approximately 10 μm thick, and the internal structure was similar to that of the ventral scale (Figure 4A,B). The dorsal scales had a thin fibrous layer approximately 1 μm thick, and the diameter of the fibers was approximately 0.3 μm (Figure 4C). Unlike at the ventral scale, there was no obvious angle of fiber arrangement at the dorsal scale.

In general, the ventral scale is approximately 2.5 times thicker than the dorsal scale, allowing for more redistribution of stress in the thickness direction. The ventral scales had more collagen fibers than the dorsal scales, and the thickness of the fibrous layer on the ventral scales was approximately 4 times greater than that on the dorsal scales. The diameter of the collagen fibers on the ventral scales was approximately 1.7 times greater than that on the dorsal scales; thus, these fibers could provide greater resistance to damage.

### 3.2. Mechanical Properties of the Scales

**Figure 5:**
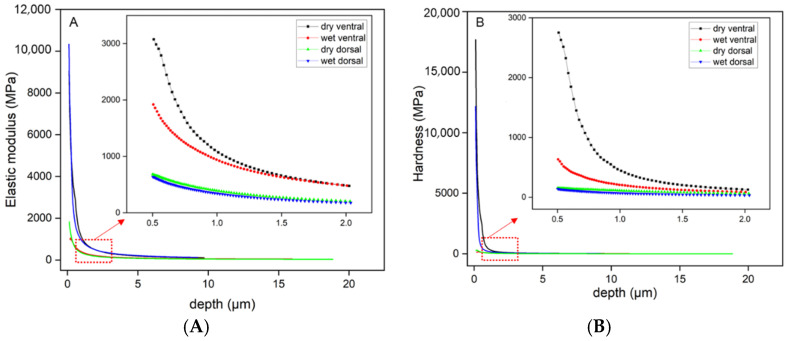
The variation in the elastic modulus (**A**) and hardness (**B**) of the ventral and dorsal scales with indentation depth under dry and wet conditions. The graph on the right is an enlarged image of 0.5–2 μm depth.

Nanoindentation experiments were carried out on the ventral and dorsal scales of the snake to determine the mechanical properties of the scales. The variations in the elastic modulus and hardness with respect to the indentation depth of the ventral and dorsal scales under dry and wet conditions are shown in Figure 5. All the data were collected through nanoindentation tests. During the entire indentation test, the elastic modulus and hardness first decrease rapidly (0–0.5 μm) and then change slowly (0.5–2 μm), as shown in the right part of Figure 5A,B. At a thickness of 0–0.5 μm, the high elastic modulus and hardness are related to the microstructure on the scale surface. However, at a thickness of 0.5–2 μm, which corresponds to the fibrous layer, the elastic modulus and hardness of the ventral scales decrease from 2500 MPa to 300 MPa and from 1000 MPa to 100 MPa, respectively. The elastic modulus and hardness of the dorsal scales decreased from ~600 MPa to ~120 MPa and from ~150 MPa to ~30 MPa, respectively. The decrease in these mechanical properties of the scales indicates that the scales consist of a hard external material and a soft internal material. Moreover, the influence of water content was more obvious on the ventral scale than on the dorsal scale. Finally, the curves tend to be flat at a thickness of less than 2 μm, which indicates that the elastic modulus and hardness of the internal tissue at a scale greater than 2 μm are similar.

**Figure 6:**
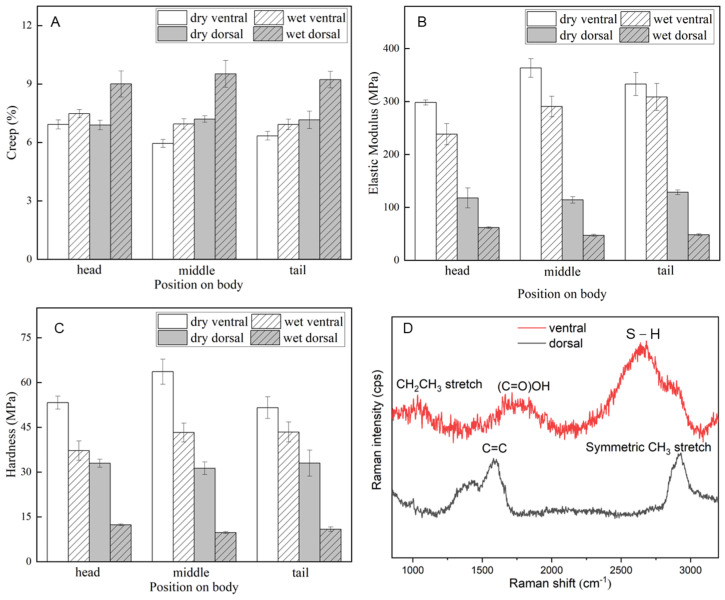
Mechanical properties and Raman spectra of scales. Relationships between the mechanical properties and position of the scales from *Dinodon rufozonatum* under the four evaluated conditions. The abscissa represents the position of the scales on the body, and the ordinate represents (**A**) the creep (%), (**B**) the elastic modulus (MPa), and (**C**) hardness (MPa). The error bars denote standard deviations. (**D**) shows the Raman spectra of the ventral and dorsal scales of the snake.

The creep, elastic modulus, and hardness of the scales were estimated by the Oliver–Pharr method based on indentation force-displacement curves. Figure 6 shows a summary of the mechanical property assessment of the location and the dry and wet conditions. As indicated in Table 1, the average value of each indicator at the head, middle, and tail positions was selected for calculation and analysis. The results for the mechanical properties (Table 1) also showed that there are differences in terms of dry/wet and ventral/dorsal properties. As depicted in Figure 6A, the creep of the dorsal scales increased noticeably under wet conditions, although it did not change appreciably under other conditions. However, as shown in Figure 6B,C, the elastic modulus and hardness of the ventral scales were higher than those of the dorsal scales, and the elastic modulus and hardness decreased under wet conditions. This may be because the ventral and dorsal scales differed in terms of their thickness and fiber content, as when wet, the laminar interstices inside the scales served as storage spaces for water molecules, changing the mechanical characteristics according to the position and environment. In general, dry and wet conditions affected the ventral scales less than the dorsal scales, which may be related to the stronger contact of the ventral scales with the ground during snake locomotion. The reduced effect of the external environment on the ventral scales could ensure their stability, helping the snake move steadily in a variety of situations.

The averaged Raman spectra and the Raman spectral characteristic peaks of the ventral and dorsal scales are shown in Figure 6D [60]. The Raman spectroscopic analysis of the ventral scales reveals prominent peaks at 1034 cm^−1^, 1714 cm^−1^, and 2682 cm^−1^, which are attributed to the CH_2_CH_3_ stretch of phenylalanine in collagen, the ν(C=O)OH vibration of amino acids such as aspartic and glutamic acid, and the ν(S–H) vibration of the amino acid methionine, respectively. However, the peaks observed in the dorsal scales are primarily located at 1582 cm^−1^ and 2928 cm^−1^, corresponding to the δ(C=C) vibration of phenylalanine and the symmetric CH_3_ stretch of proteins. The comparative Raman spectroscopic analysis indicates that both scales are composed of proteinaceous materials, as evidenced by the peak at 2928 cm^−1^. However, the ventral scales exhibit unique spectral features at 1034 cm^−1^, 1714 cm^−1^, and 2682 cm^−1^, which are either absent or significantly less intense in the dorsal scales. These differences suggest that the ventral and dorsal scales have distinct biochemical compositions, which may be related to their specialized functions or the distinct environments they inhabit within the organism.

### 3.3. Coefficient of Friction of the Scales

**Figure 7:**
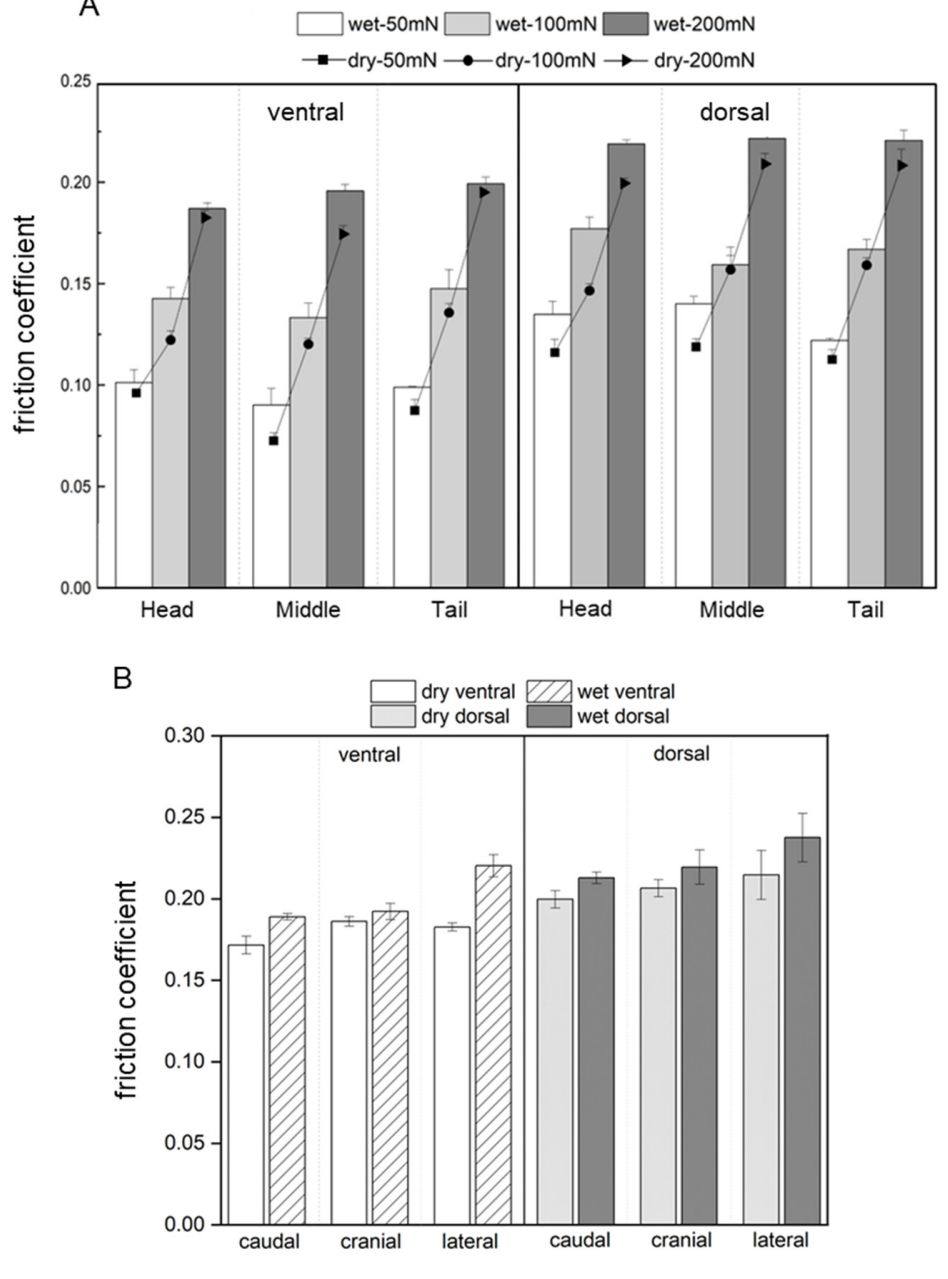
The friction coefficient of the ventral and dorsal scales under dry/wet conditions, (**A**) different forces, and (**B**) different scratch directions.

Figure 7A shows the friction coefficients of the scales subjected to three constant forces in dry and wet environments. These friction coefficients were obtained from constant force scratch tests using a Micro Scratch Tester, and the scratch directions were caudal. The results showed that the average friction coefficient of the scales in the wet environment (0.1588) was greater than that in the dry environment (0.1453), which was related to the deformation of the scales under the softening effect of water molecules. The average friction coefficient of the ventral scales (0.1383) was lower than that of the dorsal scales (0.1658). The friction coefficient of the scales did not vary significantly with the position of the scales along the longitudinal axis of the body. The friction coefficients of the different scratch speeds of the snake scale are shown in Figure S1 in Supplementary Materials. The coefficients of friction of the scales in all three directions of scratching are illustrated in Figure 7B, and the normal forces of the scratches were all 200 mN. The friction coefficients of the scales increased under wet conditions, particularly for the lateral scratches. These were connected to the snake’s serpentine locomotion and rectilinear creeping, in which the ventral scales serve as a major source of drive. The increase in the friction coefficient of scales, especially in the lateral direction, aids in providing a stronger driving force for their movements in wet conditions.

### 3.4. Scratch Morphology of Dry Scales

**Figure 8:**
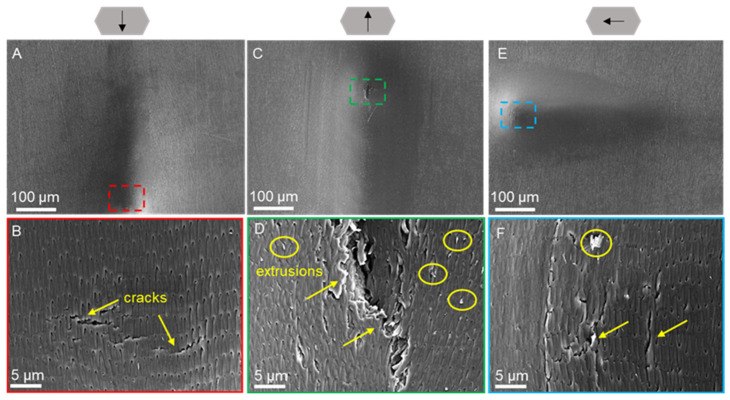
SEM images of the morphologies of the ventral scales after scratching under dry conditions at 200× (**A**,**C**,**E**) and 7× (**B**,**D**,**F**) magnification. The arrows above represent the caudal, cranial, and lateral scratch directions in (**A**,**B**), (**C**,**D**), and (**E**,**F**), respectively. The anterior part of the scale is oriented at the top of the image (**A**–**F**). Images (**B**,**D**,**F**) represent the enlarged parts of the dotted boxes in images (**A**), (**C**), and (**E**), respectively. The yellow arrows indicate cracks on the scale, and the yellow circles indicate the extrusions on the scale.

**Figure 9:**
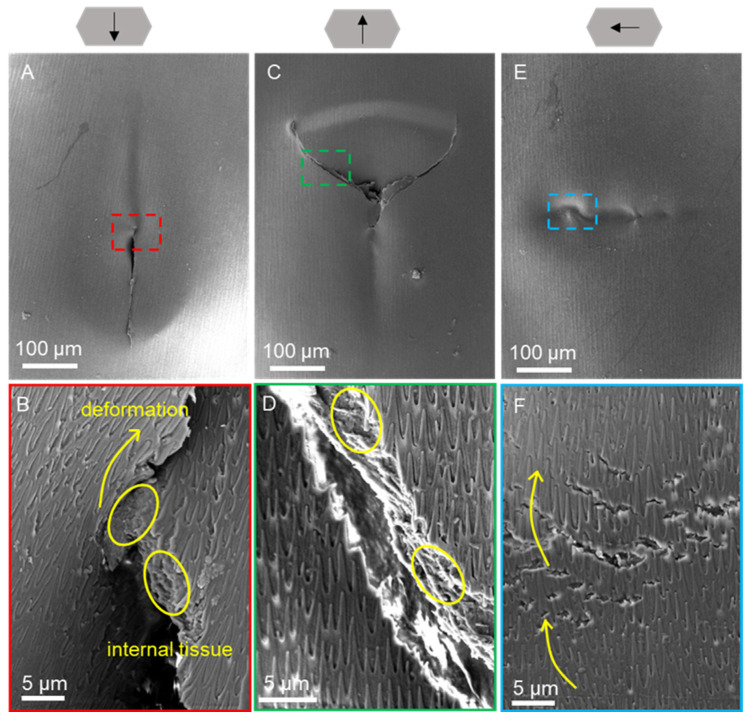
SEM images of the morphologies of the dorsal scales after scratching under dry conditions at 200× (**A**,**C**,**E**) and 7× (**B**,**D**,**F**) magnification. The arrows above represent the caudal, cranial, and lateral scratch directions in (**A**,**B**), (**C**,**D**), and (**E**,**F**), respectively. The anterior part of the scale is oriented at the top of the image (**A**–**F**). Images (**B**,**D**,**F**) represent the enlarged parts of the dotted boxes in images (**A**), (**C**), and (**E**), respectively. The yellow arrows indicate deformations of the scale, and the yellow circles indicate the inner tissue of the scale.

**Figure 10:**
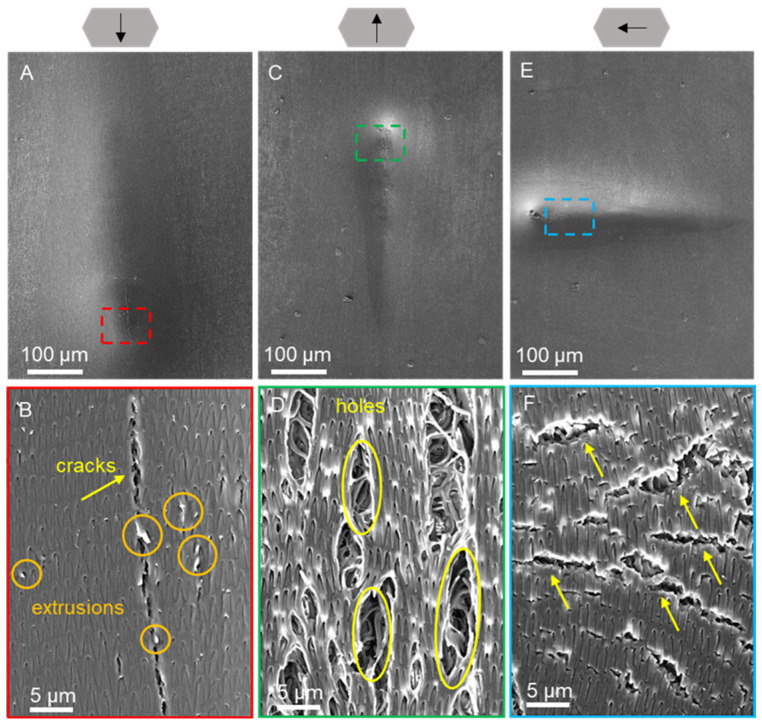
SEM images of the morphologies of the ventral scales after scratching under wet conditions at 200× (**A**,**C**,**E**) and 7× (**B**,**D**,**F**) magnification. The arrows above represent the caudal, cranial, and lateral scratch directions in (**A**,**B**), (**C**,**D**), and (**E**,**F**), respectively. The anterior part of the scale is oriented at the top of the image (**A**–**F**). Images (**B**), (**D**), and (**F**) represent the enlarged parts of the dotted boxes in images (**A**), (**C**), and (**E**), respectively. The yellow arrows indicate cracks on the scale, the orange circles indicate the extrusions and the yellow circles indicate the holes on the scale.

**Figure 11:**
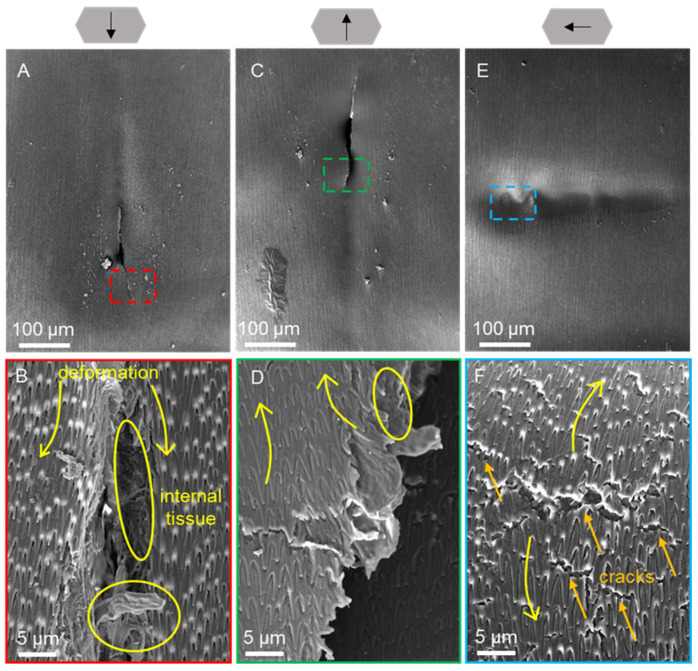
SEM images of the morphologies of the dorsal scales after scratching under wet conditions at 200× (**A**,**C**,**E**) and 7× (**B**,**D**,**F**) magnification. The arrows above represent the caudal, cranial, and lateral scratch directions in (**A**,**B**), (**C**,**D**), and (**E**,**F**), respectively. The anterior is oriented at the top of the image (**A**–**F**). Images (**B**), (**D**), and (**F**) represent the enlarged parts of the dotted boxes in images (**A**), (**C**), and (**E**), respectively. The yellow arrows indicate the deformation of the scale, the yellow circles indicate the internal tissue and the orange arrows indicate the cracks on the scale.

The morphology of the ventral and dorsal scales after progressive force scratching in dry (Figure 8 and Figure 9) and wet environments (Figure 10 and Figure 11) was observed by scanning electron microscopy for characterization. Typical friction coefficient curves are shown in Figure S2 in the Supplementary Materials.

The morphological characterization of the ventral scales after progressive force scratching in a dry environment using scanning electron microscopy is illustrated in Figure 8. The scales had depressions with cracks in all three directions (Figure 8A,C,E) and obvious cracks or openings at the end of the scratch, and the scale surface structure was deformed, delaminated, and fractured (Figure 8B,D,F). The material inside the scale is extruded (Figure 8D,F). The scale surface was torn, and the cracks developed and extended in the direction of the scale surface’s micro-convex body structure, indicating that the scale surface’s micro-convex body structure guided the cracks and could reduce the extent of scale surface damage. Compared with caudal and lateral scratches, cranial scratches resulted in more severe tearing with obvious brittle fracture (Figure 8D).

Figure 9 shows the morphological results of progressive force scratching on the dorsal scales in a dry environment. The scratches on the scales were initially undetectable, but the degree of depression increased gradually as the normal force of the scratches increased, eventually resulting in obvious cracks and delamination of the scales. In the caudal and cranial scratches, the scales were torn to a greater extent, and traces of extrusion and deformation of the scales could be observed (Figure 9A–C,F). The fractures of the cracks were neat, and the internal tissues were distinguishable (Figure 9B,D). The formation of V-shaped cracks was observed at the end of the area of cranial scratches (Figure 9C). For the lateral scratches, plastic deformation of the scales was observed, and no visible striated tears were found (Figure 9E). When the end of the scratch was magnified, many short cracks could be found on the surface of the scales (Figure 9F), and the cracks did not penetrate deeply into the base of the scales.

### 3.5. Scratch Morphology of Wet Scales

The morphological results of progressive force scratching of the ventral scales in a wet environment are shown in Figure 10. Caudal scratching revealed elongated strip cracks in the ventral scales at the end of the scratch (Figure 10B). Interestingly, ductile damage in the scratches of the wet scales was observed, in contrast to the brittle damage observed in the dry scales. As the cracks expand, the fibers begin to debond, resulting in entanglement and the appearance of irregularly distributed elliptical holes on the scale surface (Figure 10D). The intact fiber structure could locally contribute to the toughening process. According to the caudal and cranial scratches, there were more small cracks at the ends of the side scratches (Figure 10F).

Figure 11 shows the morphology of the dorsal scales after progressive force scratching in a wet environment. Long cracks were noticeable in both the caudal and cranial scratches (Figure 11A,C), while cracks were not obvious in the lateral scratches (Figure 11E). After magnification, the microstructure of the wet dorsal scales underwent evident bending and deformation due to compression during scratching (Figure 11B,D,F), while peeling, delamination, and warping of the microstructure appeared in the caudal and cranial scratches (Figure 11B,D). In addition, when the scales were scratched from a lateral direction, the crack morphology was similar to that of the ventral scale scratches shown in Figure 10F.

**Figure 12:**
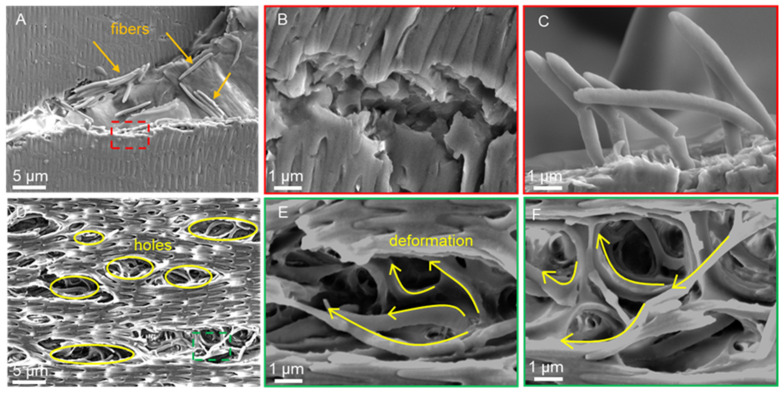
Details of the scratch morphology of the ventral scale in dry (**A**–**C**) and wet (**D**–**F**) environments at 7k× (**A**,**D**) and 30k× (**B**,**C**,**E**,**F**) magnification. Images (**B**) and (**C**) and images (**E**) and (**F**) represent the enlarged parts of the red dotted boxes in images (**A**) and (**D**), respectively. The orange arrows indicate the fibers that fell off, the yellow circles in (**D**) represent the holes produced on the surface of the scales, and the yellow arrows indicate the deformation of the fibers.

The details of the scratch morphology of the ventral scales under dry and wet conditions are shown in Figure 12. The fiber layer of the ventral scale is thicker and contains more fibers. Therefore, the fibers in the ventral scales become more prominent and easily observable after scratching. When analyzing the scratch details, we choose the ventral scales with richer details as the subjects of observation. In the dry environment, the scales underwent brittle damage with a neat fracture in the crack region (Figure 12B), and the fibers within the scales were squeezed, pulled out from the inside of the scales, and notched (Figure 12C). In contrast, in the wet environment, ductile damage was observed, and the cracked area exhibited a pore-like shape (Figure 12D), while the fibers on both the surface and inside of the scales were bent (Figure 12E,F).

**Figure 13:**
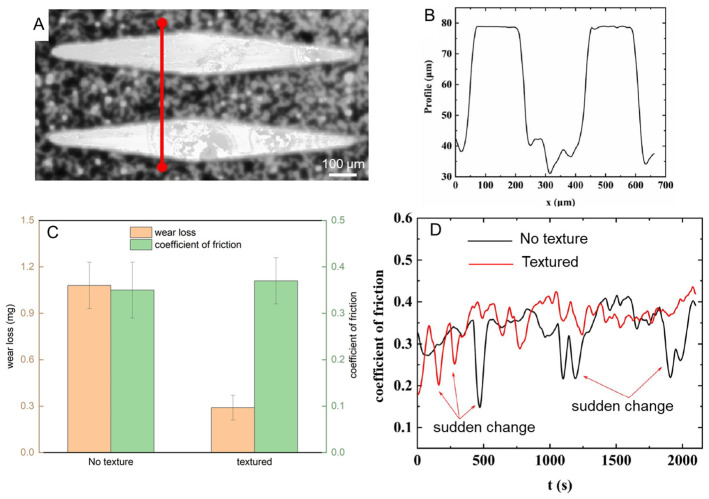
(**A**) Surface morphology of the laser-textured convex structures on a spheroidal graphite cast iron sample. (**B**) Profile of the groove section height showing the uniformity of the textured surface after laser processing and polishing. (**C**) Comparison of the wear volume between textured and non-textured surfaces after 50 min of dry friction testing. (**D**) The friction coefficients of textured and non-textured surfaces during the steady-state friction phase.

The laser-textured surfaces exhibited distinct tribological behavior compared to non-textured samples, as illustrated in Figure 13. Figure 13A shows the well-defined convex structures on the surface of the cast iron sample following laser processing, which closely resemble the microstructures observed on snake scales. The grooves and ridges of the texture provide a means of modulating contact area during friction, which plays a key role in the observed wear and friction characteristics. Figure 13B presents the profile of the groove section height, confirming the precision of the laser processing and the effectiveness of the subsequent polishing. The surface roughness was controlled to a consistent 0.2 μm, ensuring repeatable friction and wear results across the tests. In terms of wear resistance, Figure 13C compares the wear volumes of the textured and non-textured surfaces. The textured surface exhibited significantly less wear, demonstrating its potential to enhance durability during extended use. The reduced wear can be attributed to the ability of the surface texture to distribute the load more evenly and reduce localized stress concentrations. However, as shown in Figure 13D, the friction coefficient of the textured surface was slightly higher than that of the non-textured surface during the steady-state friction phase. This increased friction is likely due to the enhanced asperity contact provided by the convex structures, leading to greater interaction between the sliding surfaces. These findings suggest that laser-textured surfaces can improve wear resistance but may require further optimization to balance frictional performance.

## 4. Discussion

The mechanical properties and scratching characteristics of the ventral and dorsal scales of *Dinodon rufozonatum* were investigated under dry and wet conditions at various locations (head, middle, and tail). We propose an adaptation strategy for the snake based on our experimental results, in which the snake regulates the mechanical properties of the skin via scales, thereby regulating the friction coefficient in various living environments, particularly in terms of scratch performance.

According to nanoindentation experiments, the average elastic modulus of the ventral scales under dry conditions was comparable to that of other snake scales [13,40,54,56]. The elastic modulus and hardness of the scales were significantly lower in the wet environment than in the dry environment (Figure 6). This could be due to the fibers found in the scales, as revealed by previous studies of scale composition and cross-sectional images of the ventral and dorsal scales. (Figure 3 and Figure 4) [13,14,24,40,56]. In a dry environment, the absence of water molecules results in the formation of interpeptide hydrogen bonds between peptide chains, which alter the mechanical properties of individual and dense collagen fibers, thereby promoting a macroscopic hardening [34,61]. Furthermore, interpeptide hydrogen bonds can enhance molecular sliding resistance, limit fiber and interface sliding, inhibit fiber delamination, and help the scale resist deformation. In contrast, water molecules act as lubricants between the layers, allowing the interface to slide and deform, resulting in a large deformation at the wet scale [33,34,61,62]. The chemical composition differences between ventral and dorsal scales may significantly influence their mechanical properties and tribological behavior. The ventral scales, often subjected to different environmental and mechanical stresses compared to dorsal scales, may exhibit variations in the distribution of organic and inorganic components, such as proteins, lipids, and minerals. These compositional disparities are likely to affect the scales’ hardness, elasticity, and resistance to wear, which, in turn, would impact their frictional characteristics. As such, the investigation into the chemical composition-mechanical property-tribological behavior relationship in fish scales constitutes a promising area for future research. It would be valuable to delve into the following aspects as a research outlook.

The results of the scratching experiments revealed that wet scales have a greater friction coefficient than dry scales (Figure 7). This was related to the mechanical properties of the scales. The elastic modulus and hardness of the scales decreased in a wet environment, increasing the deformation of the wet scales. As a result, the real contact area between wet scales and the scratch head was greater than the real contact area between dry scales, and the adhesion of scales was greater, resulting in increased friction coefficients for wet scales [63,64]. In lateral undulation, the challenge for a snake is to use body bends to create forces parallel to the trunk that are strong enough to overcome the drag on the body as it moves through the terrain. Previous research on the locomotion of snakes in dry environments has demonstrated that decreasing the resistance from head to tail can enhance performance [47,65,66]. However, the increase in the friction coefficient from the head to the tail in this work in a wet environment supports subsequent research on snake locomotion in a wet environment. This change in material properties in response to the environment may reflect the adaptation of biological materials to the natural environment, which is an adaptation strategy used by organisms.

Previous studies on snake scales have concentrated on the overall morphology of the scales in a dry environment, while the morphology of the scratches in a wet environment and the microscopic fiber deformation pattern of the scales remain to be investigated. Our study complements both of these studies. Under wet conditions, the scratch morphology of the scales revealed the unique behavior of collagen fiber bending and winding (Figure 12C,D). This may be due to the continuous presence of water molecules, which give the fibrils sufficient room to migrate and elongate [67,68]. The collagen fibers in the wet scales were deflected, entangled, and pulled out, enduring the force until fracture occurred. Moreover, by developing large deformations, wet scales may dissipate the fracture energy generated by dynamic loading. Similar to fish scales [57,69,70], this failure mechanism is capable of preventing cracks from propagating to the interior, resulting in only localized damage to the scales [33,38,71]. This mechanism of scale failure in a wet environment may help protect the snake’s body and minimize damage. In contrast, there was no obvious entanglement of collagen fibers, and the scales primarily underwent delamination and fracture, indicating brittle damage (Figure 12A,B). This might be due to the absence of water molecules, which eliminates the interaction between the fiber and water molecules within the scale, hence decreasing the force between the surfaces. According to the research of Bose S. et al. on the mechanical behavior of collagen fibers, the absence of water molecules caused dry fibers to exhibit a strain-hardening response, which eventually led to brittle failure with a significant increase in resilience and toughness [67,68]. The results of Liu et al.‘s puncture test on the scales of grass carp indicate that the pattern of failure of the scales is proportional to the water content. In hydrated scales, collagen fibers twist around the puncture site, whereas shear failure occurs in the bone layer of dehydrated scales [71]. In conclusion, the water concentration of scales containing fibers has a direct effect on their ability to resist degradation. Consequently, the scales of *Dinodon rufozonatum* examined in this study exhibited distinct scratch resistance in dry and wet environments.

Scratching tests revealed that the ventral scale had a lower coefficient of friction than the dorsal scale. This is because the ventral scales have a higher elastic modulus, smaller shape variations under the same external force, and lower adhesion and intermolecular forces, resulting in less friction and a lower friction coefficient. The observed ordered lipid monolayer as a lubricating layer may be responsible for the reduction in ventral surface friction. Previous research has identified nanoscale thin lipid layers on the outermost surface of *L. california* scales. The membranes of the ventral scales were more ordered and accumulated more densely than those of the dorsal scales. On the ventral scales, an ordered “solid” lipid layer is present, whereas a semi-disordered lipid layer is present on the dorsal surface. High lipid film order promotes lubrication, minimizes wear, and enhances mechanical contact with the environment during crawling locomotion [14]. The muscles provide the primary driving force for the snake’s movement, while the scales mediate the interaction between the body and the ground, and their low friction coefficients help decrease frictional resistance and enhance movement efficiency. For scratches, the thicker ventral scale and larger diameter of collagen fibers (Figure 4) allow for enhanced stress redistribution in the thickness direction. Sasaki N. et al. showed that collagen is crucial because its viscoelasticity helps to homogenize stresses and enhances toughness [72]. According to Ji and Gao, significant deformation also aids in dissipating fracture energy under dynamic stresses [73]. When the scales were scratched, these fibers could act as an initial barrier to dissipate fracture energy through a combination of elastic and inelastic deformation, thereby preventing instantaneous damage and contributing to the uniform distribution of stresses and the reduction in stress concentration. Moreover, the extracted fibers can function as a lubricant, thereby reducing physical damage to the snake [57]. In addition, the ventral scales of the snake have the most contact and are almost always in a state of wear. Natural evolution has resulted in ventral scales that are more resistant to scratching and, therefore, help protect the snake’s body. The study of the qualities of snake scales under wet and dry conditions, as well as their scratch characteristics, can help elucidate the mechanism by which biomaterials exhibit environmental adaptation. These findings provide valuable insights for the design of high-performance bionic materials, particularly in applications that require tunable frictional properties, such as robotics, surface coatings, and medical devices [74,75,76]. For example, the ability of snake scales to adapt to different environments by altering their mechanical and frictional properties could inspire the development of smart surfaces that adjust their performance in response to external stimuli. Future research could explore how these mechanisms can be applied to the creation of adaptive materials for extreme environmental conditions.

The tribological properties of the snake scales, specifically their frictional modulation in both wet and dry environments, present valuable biomimetic applications. For example, the frictional adaptability observed in these scales could inspire the development of robotic surfaces with adaptive friction control for different terrains. Similarly, the scales’ ability to minimize wear in abrasive conditions could inform the design of protective coatings for medical tools or underwater systems where varying levels of lubrication are required. Additionally, self-lubricating surfaces, particularly in low-friction applications like surgical tools, could draw inspiration from the nanoscale lipid layers present on the ventral scales. Industrial machinery is exposed to varying environmental conditions. These coatings could mimic the scales’ ability to reduce friction under dry conditions and maintain structural integrity in wet environments. Additionally, the adaptive frictional behavior could inspire advanced robotics, where the modulation of surface friction is crucial for enhancing mobility and interaction with diverse terrains. Future research will focus on optimizing the laser-textured biomimetic surfaces to achieve a balance between wear resistance and frictional performance. Investigating the effect of texture geometry, scale, and material composition could further improve tribological properties. Additionally, the frictional behavior of these surfaces in wet environments will be explored, as the current study only examined dry conditions. Understanding how moisture impacts friction and wear will provide deeper insights into the adaptability of such biomimetic surfaces for real-world applications, particularly in industries such as robotics, underwater systems, and medical devices that require versatile, adaptive materials.

## 5. Conclusions

In summary, we examined the cross-sectional morphology of the ventral and dorsal scales of snakes, as well as the mechanical properties and scratching characteristics of the scales under dry and wet conditions. The findings of nanoindentation studies revealed that the elastic modulus and hardness of scales under wet conditions were lower than those of scales under dry conditions and that the creep rate of wet scales was greater than that of dry scales. The results of the scratching experiments revealed that the friction coefficient of wet scales was greater than that of dry scales, which could reduce the slipping phenomenon of snakes in a wet environment and improve movement efficiency. Moreover, brittle damage primarily occurred on dry scales, while ductile damage was found on wet scales, which could help the snake reduce the degree of damage when living near water. The reaction tactics of scales to dry and wet environmental variations can serve as inspiration for the development of bionic adaptive materials.

## Figures and Tables

**Table 1:** Summary of the mechanical properties and statistical comparisons of the scales under the four conditions of evaluation. The values represent the average ± standard deviation. Rows with different letters indicate significant differences.

Condition	Creep (%)	Elastic Modulus (MPa)	Hardness (MPa)
dry ventral	6.41 ± 0.22 a	331.58 ± 14.70 a	56.16 ± 3.34 a
wet ventral	7.12 ± 0.25 a	279.13 ± 21.65 b	41.28 ± 3.25 b
dry dorsal	7.08 ± 0.29 a	120.16 ± 9.85 c	32.42 ± 2.61 c
wet dorsal	9.25 ± 0.59 b	52.43 ± 1.77 d	10.98 ± 0.46 d
*p* value	<0.05	<0.05	<0.05

## Data Availability

Data are contained within the article and Supplementary Materials.

## Supplementary Materials

The following supporting information can be downloaded at: https://www.mdpi.com/article/10.3390/biomimetics9100617/s1, Figure S1: Variation of friction coefficient of scales under 200 mN constant force; Figure S2: Variation of friction coefficient of snake scales under 50–3000 mN force.
