# Supplementary material for: Effect of Surface Morphology and Internal Structure on the Tribological Behaviors of Snake Scales from Dinodon rufozonatum
Source: Biomimetics (Basel). 2024 Oct 11;9(10):617. doi: 10.3390/biomimetics9100617 (PMC11506616; doi:10.3390/biomimetics9100617)
Supplement: Supplementary file 1 [file biomimetics-09-00617-s001.zip › biomimetics-3201853-supplementary.pdf]

Supplementary File

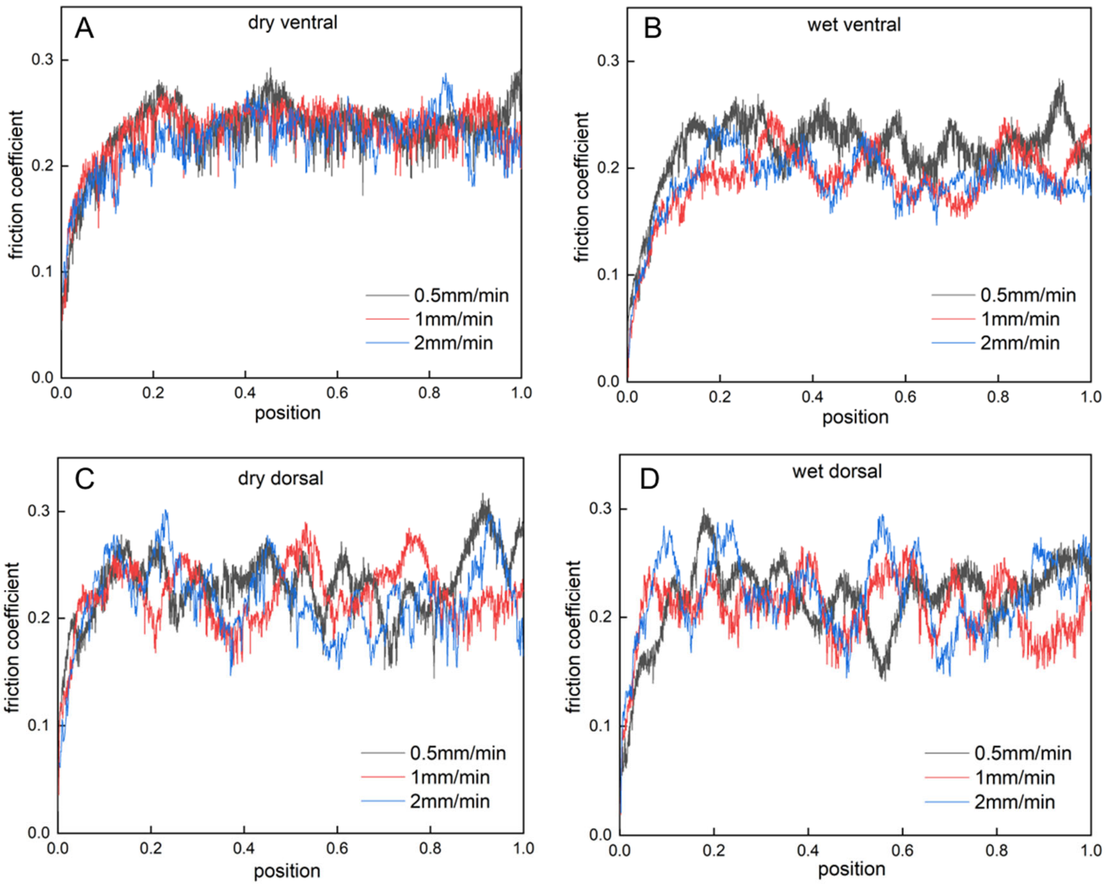

Figure S1. Variation of friction coefficient of scales under 200 mN constant force. All tests are conducted under 0.5, 1, and 2 mm/min scratch speeds in the caudal direction. (A) dry ventral scales; (B) wet ventral scales; (C) dry dorsal scales; and (D) wet dorsal scales.

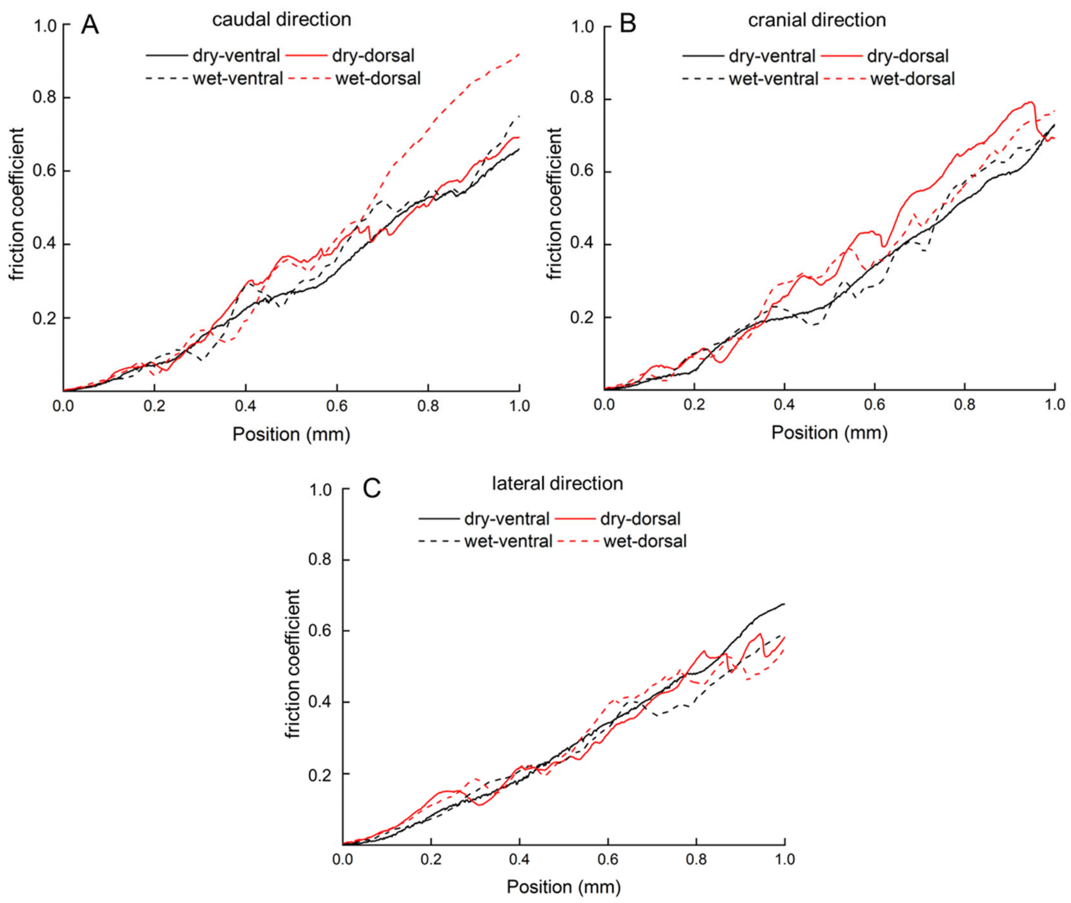

Figure S2. Variation of friction coefficient of snake scales under 50-3000 mN force. (A) caudal direction; (B) cranial direction; and (C) lateral direction.
